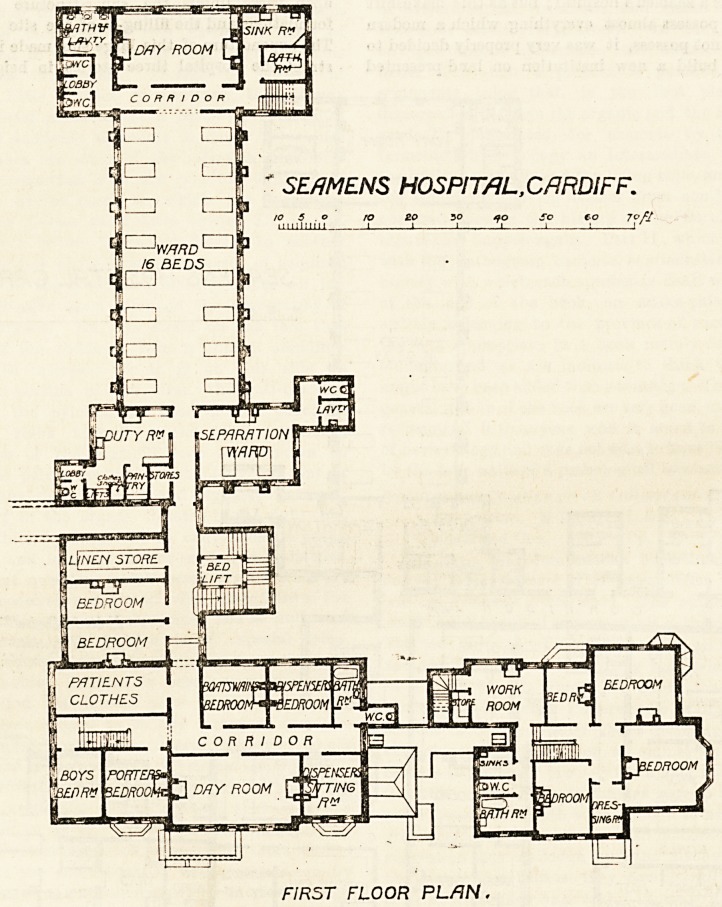# Seamen's Hospital, Cardiff

**Published:** 1903-09-19

**Authors:** 


### HOSPITAL ADMINISTRATION.

### CONSTRUCTION AND ECONOMICS.

### SEAMEN'S HOSPITAL, CARDIFF.

FOR many years past an old man-of-war, the *Hamadryad*, has been used as a seamen's hospital; but as this makeshift must inevitably possess almost everything which a modern hospital should not possess, it was very properly decided to abandon it and build a new institution on land presented by the Marquis of Bute. This site was a piece of marsh land close to the old ship, and has the advantages of being near the shipping; of being open on three sides, and of having good roads to it. The site, however, had the disadvantage of being, in its natural state, too soft to carry any portion of the buildings, so that it was necessary to sink shafts from six to nine feet apart, and fill these in with concrete. The piers formed in this way are spanned by lintels, 4 feet by 4 feet, formed of cement concrete, and upon these lintels the superstructure is raised. These foundations and the filling-in of the site cost about £5,500. The expenditure of this large sum made it desirable to construct the hospital three stories in height; and the substructure is sufficiently strong to carry another story should that be necessary at any future time.

GROUND FLOOR PLAN.

The ground floor consists of the doctor's house, having a private entrance, and also an approach from the hospital corridor. Close to the latter are the accident ward, the dispensary, the consulting-room, and the waiting-room. Close to the waiting-room are the office and the main entrance. Directly in front of the entrance is one of the communicating corridors, and the other is placed at right angles to it, thus forming two sides of a square, within which are the kitchen and laundry departments, these being separated from each other by a yard. Near the junction of the two corridors is the main staircase, having in its centre a lift for beds. The operation theatre is at the other end of the corridor. It is lighted by four windows, three of them being to the north, and is provided with an anæsthetic room, a changing room, and a sterilizing chamber. Near the theatre is the Roentgen Ray room. There is a small day-room, having three large windows facing south. So far as patients' accommodation goes, the first floor is practically a replica of the ground floor, and hence need not be described.

The ward floors are of concrete, supported on iron girders, and will be finished in terazzo. This flooring is cold to the feet of those employed in the ward, but it ensures a smooth, non-absorbing surface, provided it can be kept from cracking. Unfortunately, experience shows that this cracking of the surface is very liable to take place, and it may be doubted

FIRST FLOOR PLAN.

isolation ward entered only from the road, where an infectious case can be temporarily kept.

The main ward has its axis north and south. At its north or corrider end it has a duty-room overlooking the ward, and stores, pantry, clothes shoot, and nurses' closet are close to it. Opposite to the duty-room is a separation ward for two beds. It has cross ventilation, and has a small lavatory and a closet attached to it. Between the duty-room and the separation ward is the entrance to the main ward. This ward contains 16 beds, and each bed has a window on both sides of it. At its south end a corridor runs the whole width of the ward. At one end of this are the bath-room and an emergency staircase, and at the other are the closets and another bath-room with lavatory. Between these is a whether it is better in the end than a well-laid thoroughly-seasoned floor of teak; but it is the fashion at present, and possibly another ten years will settle the point.

The walls and ceilings will be finished smooth with cement, then painted and varnished.

Heating will be by a low-pressure hot-water system, and this is admirable as an auxiliary, but not as an exclusive system, which should always be radiant heat from an open fire. Natural ventilation will be depended on, the fresh air being admitted on the floor levels and extracting flues on the ceiling levels.

The hospital is intended for accidents only, and most likely male nurses will be employed.

The component parts of the buildings are well grouped, and the planning for the most part is decidedly good; but, had we been designing the large wards, we should have omitted the corridor at the south end, have had the dormitory opening direct into the day-room, and have approached the bath-room and closets by short passages private to them. The cost has been defrayed partly by subscriptions and partly by bequest of the late Marquis of Bute, who took much interest in the work. The foundation-stone was laid by the present Marquis about a year since. The total cost will be about £27,050.

The architect is Mr. E. W. M. Corbett of Cardiff.

## Figures and Tables

**Figure f1:**
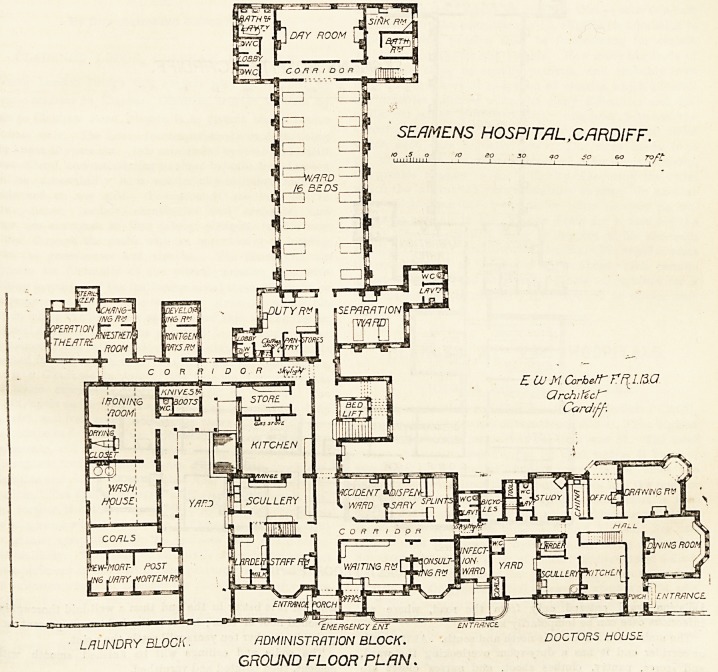


**Figure f2:**